# Research Advances of Prevention and Control of Hydrogen Sulfide in Coal Mines

**DOI:** 10.1155/2019/8719260

**Published:** 2019-04-18

**Authors:** Qigen Deng, Jingping Yin, Xifa Wu, Tao Zhang, Hao Wang, Mingju Liu

**Affiliations:** ^1^School of Safety Science and Engineering, Henan Polytechnic University, Jiaozuo 454003, China; ^2^State Key Laboratory Cultivation Base for Gas Geology and Gas Control (Henan Polytechnic University), Jiaozuo 454003, China; ^3^Collaborative Innovation Center of Coal Safety Production of Henan Province, Jiaozuo 454003, China

## Abstract

Sudden emission and casualty accidents caused by abnormal enrichment of hydrogen sulfide (H_2_S) in coal mines are becoming frequent increasingly, causing major casualties and environmental pollution. Scholars in various countries have developed various measuring devices for hydrogen sulfide content in coal and rock formations and their calculation methods. The existing prevention and control technologies of H_2_S in coal mines were summarized in various countries. According to the distribution characteristics, occurrence modes, and emission forms of H_2_S in coal mines, the prevention and control technologies of H_2_S in coal-bearing strata, airflow in tunnel, and underground water body are mainly introduced. Analyzed the effects of different ventilation systems on prevention and control of H_2_S, which include conventional ventilation system, partial homotropal ventilation system, and full homotropal ventilation system. The methods used mainly include neutralization by injecting alkalizer through drilling in coal seams with high pressure, spraying alkalizer in tunnel, attenuation by increasing wind amount, changing the ventilation method, pumping, dredging, and blocking the water that contains H_2_S as well as comprehensive prevention and control method. The basic agents adopted mainly include sodium carbonate (the mass percentage concentration is about 0.5% ~ 3.0%) and sodium bicarbonate solution, and some basic solution is added by an additive, such as surfactant, Fenton reagent, sodium dodecyl benzene sulfonate, sodium hypochlorite, or chloramine-T. The treatment effect and the main problems of each prevention and control technology are analyzed, and a comprehensive method of prevention and control techniques of H_2_S in coal mines is proposed. According to current technological level as well as the cost, the effective prevention and control techniques of H_2_S should take the occurrence, distribution, and emission forms of H_2_S in coal mines as well as the content into consideration.

## 1. Introduction

Accidents and potential hazards caused by abnormal enrichment of hydrogen sulfide (H_2_S) in coal and rock layers have emerged at home and abroad and are increasing rapidly during coal mining process. Such disasters have occurred in the former Soviet Union, Poland, Ukraine, Russia, India, and Australia. In China, such accidents are particularly serious. In recent years, more than 50 times H_2_S suddenly emerged in more than 10 provinces and autonomous regions in China, causing many deaths and injuries, as well as environmental pollution [1]. In addition, there are quite a few abnormal mines with low concentration of H_2_S, due to the low concentration of H_2_S, which have not caused widespread concern; however, it has caused irreversible damage to the central nervous system and respiratory system of underground workers, and some miners have serious sequelae; it poses a great threat to their physical and mental health. In addition, the high-sulfur coal is widely distributed in China. With the improvement of mining technology and the increase of mining depth, the possibility of high H_2_S in high-sulfur coal is bound to increase. H_2_S is a colorless, neurotoxic substance with a stinking egg flavor that primarily harms the central nervous system and respiratory system [1, 2]. The US Department of Health (1975) allows the concentration of H_2_S in the air ACGIH TLV-TWA to be 14 mg/m^3^ (9 ppm) and short-term exposure to be 27 mg/m^3^ (18 ppm); the Japanese Industrial Hygiene Society (1975) and Germany both determined it to be 15 mg/m^3^ (10ppm); and the maximum allowable concentration in coal mine air in China is 6.6ppm (volume fraction 0.00066%). Judging from the current situation of coal mining in various parts of the world, the concentration of H_2_S in many coal mines is from small to several ppm, up to several thousand ppm; the concentration of H_2_S in some sections, roadways, or mining faces is over the standard for a long time and seriously. Therefore, exploring prevention and control technology of H_2_S in coal mine is an urgent and important issue. The comparison of the international H_2_S limits to human body are as shown in Table 1.

## 2. Prevention and Control Technology of H_2_S in Coal Seams

H_2_S is mainly distributed in coal-bearing rock formations and groundwater bodies in coal mines. According to the distribution characteristics, occurrence forms, and emission patterns of H_2_S in coal and rock layers, the prevention and control techniques of H_2_S can be generally divided into the following categories.

### 2.1. Prevention and Control Technology of Hydrogen Sulfide in Coal-Bearing Strata

**Figure 1:**
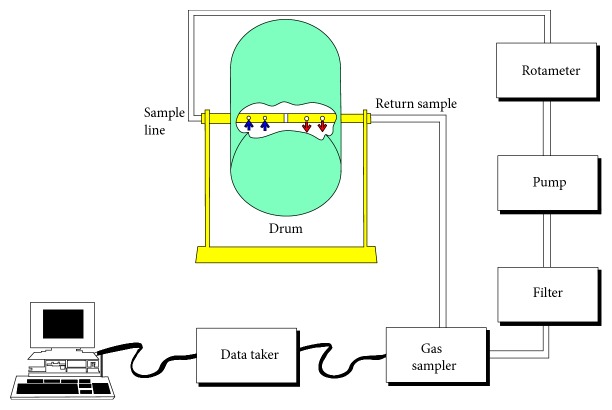
Drum tumbler system.

In the prevention and control of hydrogen sulfide in coal-bearing strata, the content and distribution characteristics of hydrogen sulfide in coal rock formation must be determined first. A drum tumbler system was developed to determine the H_2_S content of coal (Phillips et al., 1990) [6]. A modified design with the ability to constantly sample gas during coal breakage was developed (see Figure 1) [7]. The system rotates a 255 litre drum constructed from high density polyethylene, end to end about a central stainless steel shaft. The drum tumbles a weighed sample at 20 rpm for 60 revs. The period of rotation is selected to produce coal breakage representative of the size of coal on the armoured face conveyor. The sample is sized after testing. The test is used to determine the volume of H_2_S released into the atmosphere from a given sample under controlled conditions.

**Figure 2:**
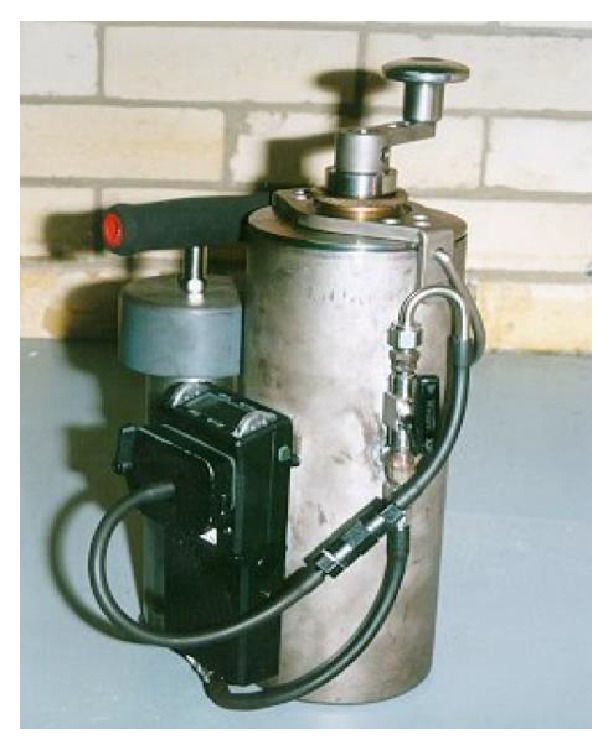
Portable gas analyzer.

A portable gas analyzer was designed (see Figure 2) [7]. The portable gas analyzer is a portable coal sample crusher that is intrinsically safe and can be taken directly to the mining face for in situ coal seam sample testing for gases. The construction of stainless steel was chosen to avoid corrosion and to allow use in gassy underground coal mine areas.

**Figure 3:**
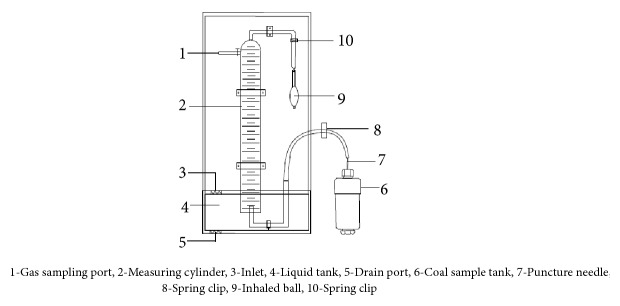
Desorption device in underground.

**Figure 4:**
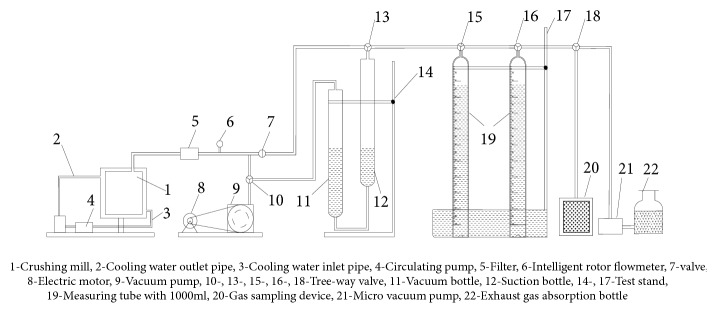
Hydrogen sulfide content measuring device of coal seam.

For the determination of the volume content of hydrogen sulfide in tons of coal, Mingju Liu et al. [8] proposed and invented a coal seam hydrogen sulfide content determination device, which mainly consists of five parts: desorption device in underground, desorption device in ground, crushing and degassing device, balance, and gas chromatography. The content of hydrogen sulfide is divided into two parts: desorption of atmospheric pressure and desorption of pulverization; the atmospheric pressure desorption of hydrogen sulfide includes loss, desorption in underground and ground. The device is shown in Figures 3 and 4.

The content (*W*) of H_2_S tested includes atmospheric pressure desorption amount *W*_1_ in underground, the gas loss content *W*_2_, the desorption amount *W*_5_ in ground, and the contents *W*_3_, *W*_4_ dissolved in the water body. The test device fully considers the physical and chemical properties of hydrogen sulfide and gives full consideration to the process of device fabrication, material selection, and gas content testing and calculation, ensuring the accuracy and reliability of the test. The hydrogen sulfide content of the coal seam can be directly and accurately determined, the engineering quantity is relatively small, the measurement cost is low, the measurement period is short, the measurement reliability and the accuracy rate are high, and a large number of measurement points can be measured, but with insufficient testing accuracy.

**Figure 5:**
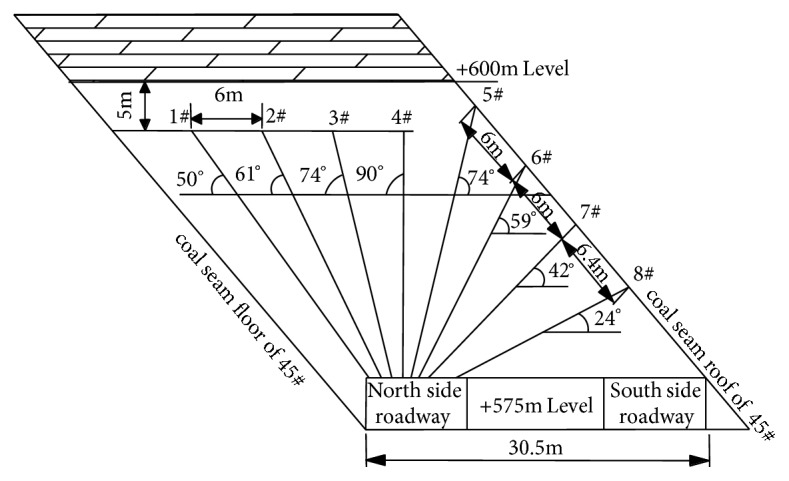
Schematic diagram of drilling and controlling H_2_S in prefilled absorbing liquid of steeply inclined thick coal seam.

Zheng Sanlong et al. [9] aimed at the high hydrogen sulfide concentration of caving top coal in fully mechanized coal face of steep inclined thick seam in the Wudong Coal Mine and adopted prefilling liquid technology to prevent and control hydrogen sulfide in advanced working face. It is determined that the water injection pressure of the coal wall of the coal seam of the inlet wind tunnel was 4.0 MPa, the time of injection of alkali was 340 min, the single-hole water injection was controlled at about 7.8 m^3^, the flow rate of alkali injection was 20 L/min, and the wetting agents added to the water injection were 1.8% and 0.2%, respectively. The wetting radius of coal body after lye injection can reach 2 m~25 m. 8 injection holes were arranged around the coal wall of the inlet section, the spacing of the drilling holes was 4 m, and the hole sealing depth ranges from 4.0 m to 4.8 m. After treatment, the mean value of hydrogen sulfide diffused to the sidewalk of the scaffold discharged coal, the support coal was reduced from 202.6 ppm to 66 ppm, and the average hydrogen sulfide in the downwind scraper conveyor was reduced from 1340 ppm to 340.8 ppm. The drill holes arrangement on working face is shown in Figure 5.

According to the emission rule of H_2_S in E902 working face of a mine in Shanxi province, Yuan Xinpeng et al. [10] derived a relationship of alkali injection parameters.

Alkali injection quantity *m*_0_: Complete neutralization of hydrogen sulfide in tons of coal requires the quality of baking soda *m*_0_:

(1) $$\begin{array}{c} m_{0}=\frac{3\;750k_{1}Q_{h}C}{vMpL_{c}} \end{array}$$

where *k*_1_ is the reserve coefficient of alkali injection; *Q*_*h*_ is the return air volume, m^3^/min;* C* is the volume fraction of hydrogen sulfide, %; *v* is the recovering speed of the shearer, m/min;* M* is the mining height, m;* ρ* is coal density, t/m^3^; and* Lc* is the drum depth, m.

The alkali injection drilling control range of completely neutralized hydrogen sulfide requires the baking soda mass *m*_1_ to be

(2) $$\begin{array}{c} m_{1}=\frac{3\;750k_{1}{bL_{a}Q}_{h}C}{vL_{c}} \end{array}$$

where* b* is the drilling pitch, m, and *L*_*a*_ is the drilling length, m.

Water injection amount *V*_*w*_: The lye is injected into the coal seam with water as the solvent. According to the mass fraction of the alkali liquor, the control range of the alkali injection drilling is completely neutralized and the water injection amount required is

(3) $$\begin{array}{c} V_{w}=\frac{\left(1-n\right)m_{1}}{np_{w}}=\frac{3\;750k_{1}bL_{a}Q_{h}C}{vL_{c}}\times \frac{\left(1-n\right)}{np_{w}} \end{array}$$

where* n* is the alkali mass fraction, %, and *ρ*_*w*_ is the density of water, kg/m^3^.

By injecting NaHCO_3_ into the coal seam after 51 h, it was found that the alkali injection had a radius of 6 m, and the volume fraction of hydrogen sulfide in the range of 6 m fell below 0.0015% on average, with a drop of 40% to 75%.

Zhao Yisheng et al. [11] treated H_2_S in No. 9 coal seam of Xiqu Coal Mine by using deep-hole pulsed dynamic pressure injection method. After drilling and sealing, the pulsed high pressure (4.0 MPa~5.0 MPa) was used to inject a sodium carbonate solution with a concentration of 1.13% into the single hole of the coal seam for 2 d~3 d. The average tons of coal injection in the positive coal seam were 10.65 kg, the amount of alkali injected was 0.12 kg, and the hydrogen sulfide was reduced from a maximum concentration of 350 ppm to an average of 3.6 ppm after treatment.

The 201 working face of Tingnan Coal Mine in Changwu city of Shanxi province uses high pressure grouting pump to carry out preharvest coal body lime-slurry water through deep-hole presplitting blasting, and combines ventilation and drainage, hydrogen sulfide pumping, and other measures to treat hydrogen sulfide in coal, achieving the desired effect [12].

Sun Weiji et al. [13] used a sodium bicarbonate solution with a mass concentration of 0.5% to 1.0% and arranged a hole with a hole spacing of 8 m, a hole diameter of 75 mm, and a hole depth of 60 m to treat hydrogen sulfide by injecting liquid into the advanced working face. The injection amount of one hole is 2.5 m^3^ ~ 2.75 m^3^, and the effect of hydrogen sulfide is better when the advanced working face is 25 m~50 m.

According to the H_2_S gas source and gush source characteristics of the B_1+2_ coal seam in the east wing of the alkali ditch coal mine in Wudong mining area, combined with the occurrence of the steeply inclined coal seam, Cheng Yuanxiang [14] adopted three measures for the simultaneous treatment of hydrogen sulfide, namely, the upper negative pressure extraction, the middle injection alkali, and the negative pressure, achieving good results.

In the process of alkali injection, when sodium carbonate or sodium hydrogen carbonate is used as the absorption liquid, the chemical reaction that may occur is as follows.

(4) Na2CO3+H2S→NaHS+NaHCO3

(5) NaHCO3+H2S→NaHS+H2O+CO2

(6) Na2CO3+CO2+H2O→2NaHCO3

The hydrogen sulfide reaction product is HS^−^, which is unstable in nature. It often dissipation from the solution or the reaction product and then diffuses again to the coal rock mass or air under the action of coal seam mining, gas drainage, and water flow. Due to the different degrees of metamorphism, composition, and development of coal in different mining areas, the wetting effect of coal is quite different, and the addition of surfactant can effectively reduce the surface tension of the absorbent and increase the penetration radius of the liquid, thereby improving the removal efficiency of adsorption of H_2_S inside the coal body. Therefore, while using an alkaline reagent as the absorption liquid, an effective and stable additive or surfactant [15] is often added to increase the absorption efficiency of hydrogen sulfide in the coal body, and the H_2_S is oxidized to elemental sulfur or sulfur compounds with higher valence and promotes the reaction in the positive direction.

**Figure 6:**
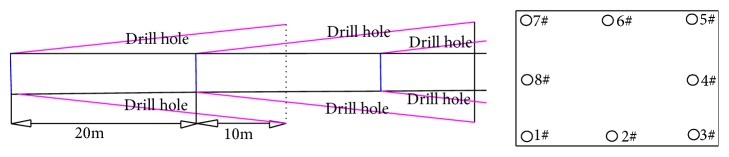
Profile chart of alkali injection hole and alkali injection excavation process drawing in heading face.

Lu Zhigang et al. [16] uniformly arranged 3 layers of 8 deep 30 m alkali injection holes along the axial direction of the roadway around the circumference of the driving working face. Inject 30 m of alkali along the axial direction, dig 20 m, leave a safe distance of 10 m, and then grout and dig circularly as shown in Figure 6. The lye formulation used was as follows: a sodium carbonate mass percentage concentration of 1.0%, a sodium dodecyl benzene sulfate and a sodium hypochlorite concentration percentage of 0.1%. It can effectively ensure a certain distance, lye wet safety curtain around the roadway, and reduce the emission of H_2_S in the tunneling.

Liang Bing et al. [17], based on the H_2_S occurrence characteristics and alkali injection parameters in Tiexin Coal Mine, studied the prevention and control of H_2_S in the mine using numerical simulation and field experiment. The flow of lye in the coal rock fracture is set to satisfy the N-S equation, the flow in the microhole satisfies the Brinkman equation, and the change in the reaction substance satisfies the convection-diffusion equation. The results show that the influence range of alkali injection increases with the increase of alkali injection time. The injection of alkali for 18 h from the hole were 7 m~9 m, the volume fraction of H_2_S decreases by more than 51.3%, and the coal seam H_2_S is almost completely neutralized within 7 m. According to the simulation results, the test was carried out with an injection hole spacing of 8 m. The volume fraction of the return air flow H_2_S after injection of alkali was 5.6 ppm, indicating that the simulation results can guide the injection of alkali in the coal seam and prevent hydrogen sulfide.

A study was undertaken to determine where H_2_S was released during mining [3–5]. Data was collected from a series of H_2_S monitors placed along the face and in the longwall conveyor roadway.  Table 2 shows the proportion of H_2_S released from each source.

From Table 1, it can be found that the sources of most of H_2_S come from shearer. Phillips [6] found that the total H_2_S content of coal could be greater than 500 litres per ton, but only between 2% and 10% of total H_2_S is released during mining.

**Figure 7:**
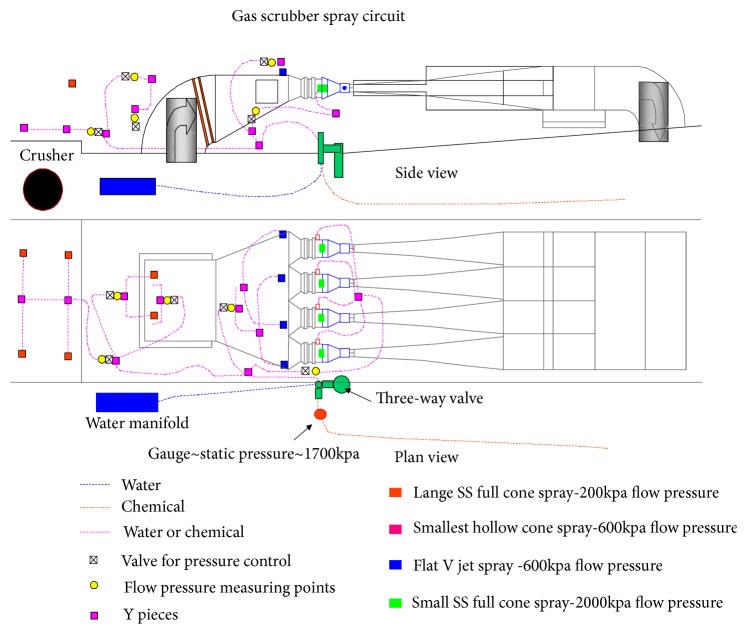
H_2_S scrubber spray circuit.

**Figure 8:**
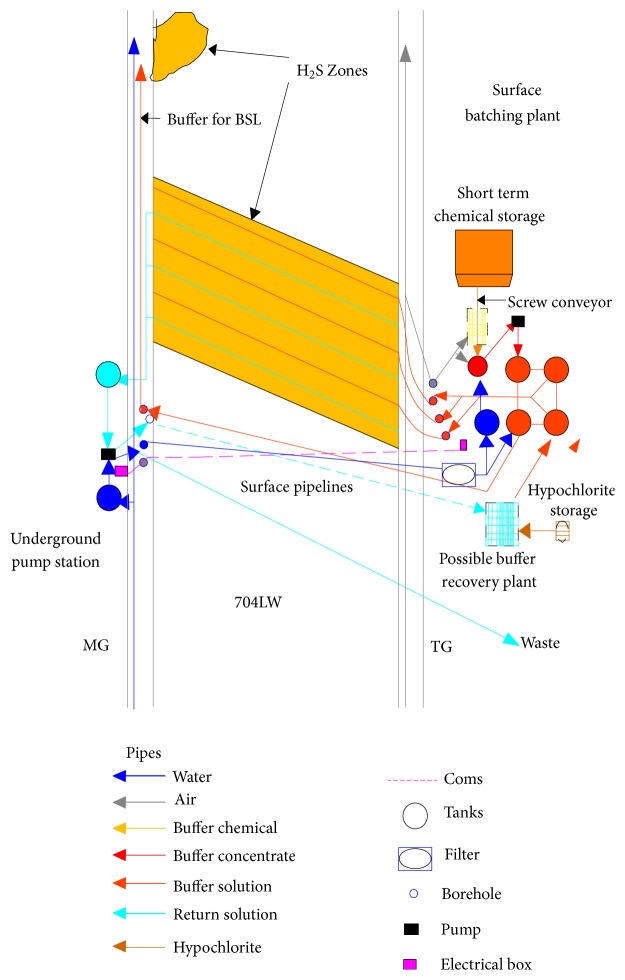
Longwall infusion infrastructure.

A venturi scrubbing unit using buffer solution was tried on the crushing unit to reduce dust and gases entering the face ventilation. When the H_2_S monitor on the face read a H_2_S gas level of more than 10 ppm, the sprays in breaker stage loader and scrubber switched from water to buffer solution. This saturation process eliminated up to 70 percent of the H_2_S gas emissions at the breaker stage loader. An illustration of the system is shown in Figures 7 and 8 [3, 18–20].

After the zone of H_2_S was identified, the appropriate infusion design was determined. A prevention system and monitoring equipment were established, as shown in Figure 8 [5, 18, 19]. The chemical buffer chosen was sodium bicarbonate. The infusion process was carried out as follows: infusion fluid was prepared on the surface by mixing of sesquicarbonate and water. The mixture flowed to the underground manifold to be distributed into the odd number boreholes under static head pressure. The fluid filled the boreholes under pressure. As each borehole end was blocked by a high pressure packer, the fluid was forced to flow through the coal seam (cleats, matrix, and pores). The fluid migrated toward the even number boreholes. As the fluid flowed through the seam, H_2_S from the coal passed into solution. The H_2_S laden fluid flowed from the even boreholes into an underground tank for pumping to the surface. On the surface neutralization occurred and sulfides present in the fluid broke down quickly.

### 2.2. Hydrogen Sulfide Prevention and Control Technology in Tunnel Airflow

In recent years, the prevention and control of hydrogen sulfide in the flow of mine tunnels are usually carried out by using series ventilation, pressure equalization ventilation, and increasing air volume, changing ventilation, or using lye chemical neutralization.

**Figure 9:**
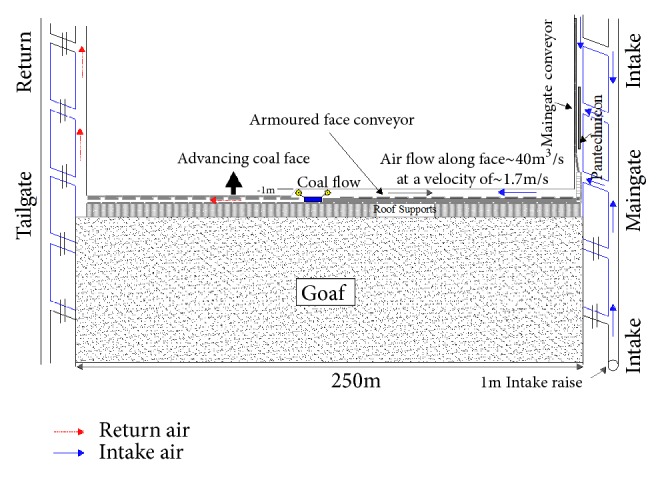
Conventional (antitropal face and belt airflow) ventilation system.

The influence of various ventilation methods on the prevention and control of hydrogen sulfide was analyzed by Gillies et al. [21]. In a longwall panel conventional or U configuration ventilation system, fresh air enters the longwall face through the maingate roads and contaminated air leaves the face through the tailgate road, as seen in Figure 9. This approach controls H_2_S by diluting and carrying the liberated gas directly from sources to the return airway. The main disadvantage of this system is that intake air may be contaminated with H_2_S gas released from the coal on the panel belt or during passage through the breaker and stage loader before reaching the longwall face.

**Figure 10:**
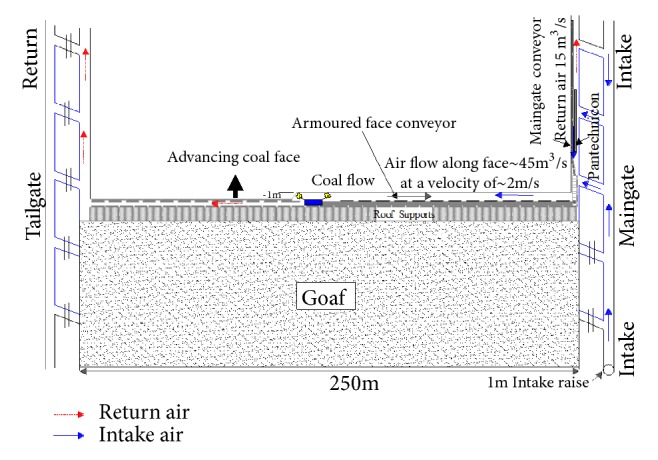
Partial homotropal ventilation system.

If an increase in the ventilation quantity does not reduce the H_2_S level to a safety threshold, the partial homotropal ventilation system should be employed, as seen from Figure 10. An axillary ventilation system can be set up around the stage loader and breaker sources to reduce the effects of this contamination on the system. The belt road was changed to return from intake, and the intake air delivered from the maingate A Heading was split between the face and the belt road. Three venturi fans (compressed air blowers) were mounted on the breaker stage loader to remove the H_2_S gas liberated during the breakage and transfer coal from face to panel conveyors. The fans sucked air from above the breaker and were connected to a ducting running along the breaker stage loader and linked to a ducting hung over the belt and dumping contaminated air into return. The system prevented a high quantity of liberated gas from contaminating the intake air and reduced the level of H_2_S at the maingate dramatically.

**Figure 11:**
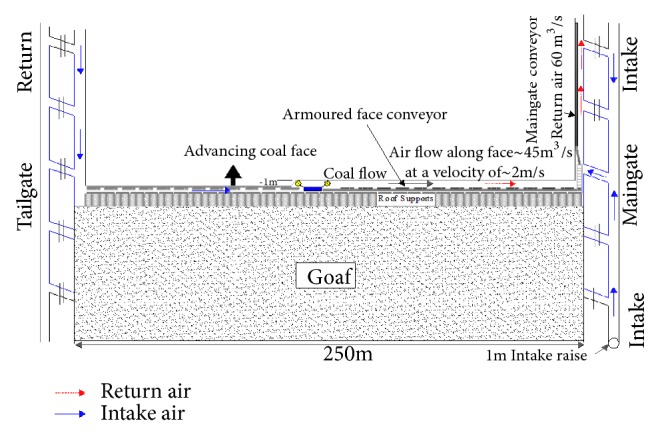
Full homotropal ventilation system.

If increasing the panel ventilation quantity or adoption of partial homotropal ventilation is not sufficient to reduce the level of H_2_S to a safety threshold, then a full homotropal ventilation system should be employed. Under this system the panel airflow direction is reversed in order to eliminate the problem of intake air being contaminated by the sources of H_2_S as seen in Figure 11. In this system, all the sources of H_2_S stay on the return side and uncontaminated fresh air is supplied to the face. The belt road becomes the sole panel return.

**Figure 12:**
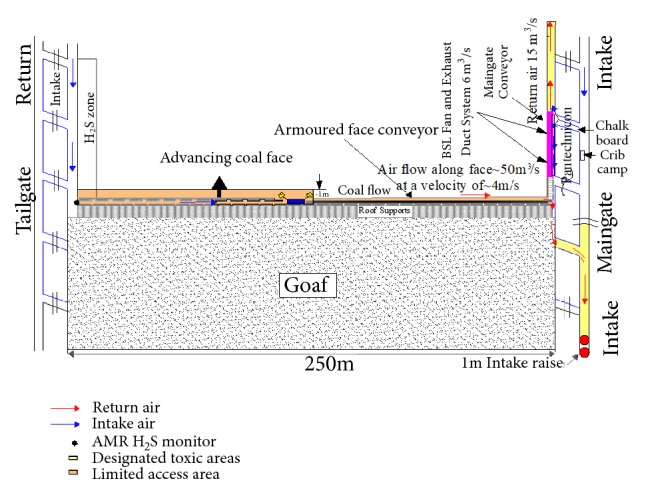
Alternative full homotropal system.

It is believed that these levels can only be achieved with acceptable production levels unless an alternative full homotropal ventilation system at the longwall face with some air passing through a back return is used. The alternative full homotropal system is as shown in Figure 12.

**Figure 13:**
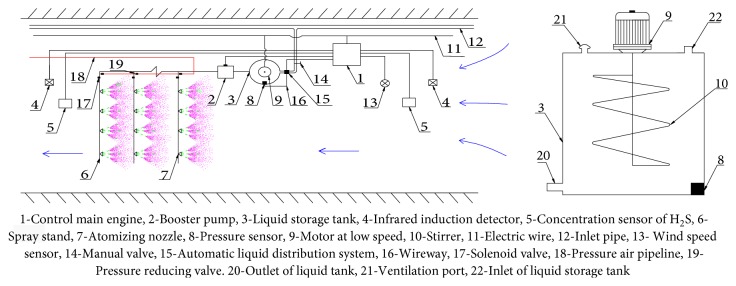
Prevention and control technology scheme of hydrogen sulfide in the air of tunnel.

Deng Qigen et al. [22] proposed and established a device and method for automatically removing hydrogen sulfide in roadway airflow. According to the H_2_S concentration and air volume in the tunnel airflow, the automatic equipment of the chemical concentration and the automatic quantification of the spraying can be realized, thereby safely and efficiently solving the problem of removing hydrogen sulfide in the tunnel airflow and effectively saving cost and labor input. The device is shown in Figure 13 [22].

Through the air pressure pipeline, a double-flow atomizing nozzle is selected; one head is introduced into the water, and the air with a certain pressure is introduced into the other head, and the air in the nozzle chamber collides with the alkali liquid to generate a fine water mist (dry mist) having a particle diameter of less than 10 *μ*m. It is beneficial to the diffusion of lye in the wind flow, increasing the air contact area, prolonging the contact time, and promoting the absorption neutralization of H_2_S gas. Three rows of nozzles are arranged, with four in each row, and the nozzles are distributed at 90°. The spacing of each row of nozzles can be adjusted according to the section size of the roadway, the concentration of hydrogen sulfide, and the speed of the wind.

Yu Yujiang et al. [23] fundamentally designed a chemical and equipment for the rapid removal of H_2_S from high-sulfur coal mines. Sodium carbonate is used as the basic absorbent, and chloramine-T is used as the catalytic oxidant. The solution is prepared according to the sodium carbonate mass fraction of 0.15% to 0.3% and the chloramine-T mass fraction of 0.3% to 0.5%. The unit is capable of automatic spraying with a spray volume of 2.5 L/min and a mist distance of 15 m to 20 m.

**Figure 14:**
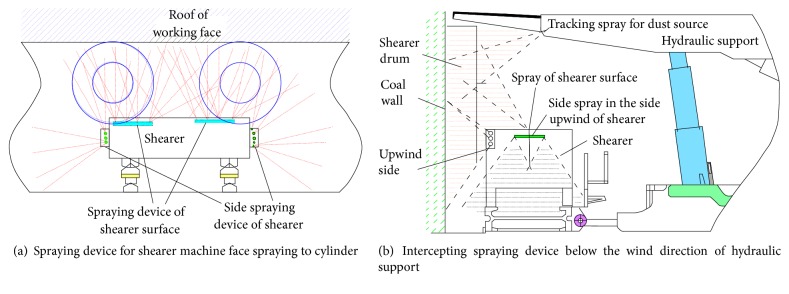
Spraying absorption liquid to control the coal mining machine to disturb the hydrogen sulfide system.

Zhang Ge [24], combined the emission characteristics of hydrogen sulfide during the extra-thick caving of coal in the north mining area of Wudong Mine, proposed and implemented the prevention and treatment of the absorbing liquid sprayed at the coal discharge port with right opposite support and intercepting and a spraying absorbent at the downwind side of the coal discharge port; the system diagram is shown in Figure 14 [24]. The spray pressure is set to 8.0 MPa, the concentration of the absorbed liquid is 0.9%, the flow rate of the spray device sprayed toward the drum is about 120 L/min, the three flow intercepting spray devices are arranged under the coal inlet, and the single spray rate is 20 L/min. Through the treatment, the hydrogen sulfide of the shearer driver was reduced from 77 ppm to 12 ppm, and the hydrogen sulfide of the rear scraper conveyor tank was reduced from 1340 ppm to 186 ppm [24].

**Figure 15:**
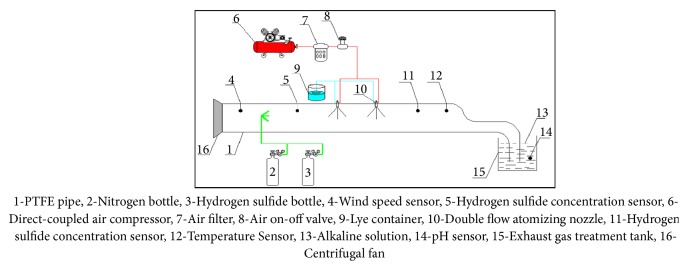
Experimental device for removing hydrogen sulfide gas from wind flow.

Deng Qigen [25] built a set of H_2_S absorption simulation experiment system in the wind flow, as shown in Figure 15 [25]. The factors affecting the removal of hydrogen sulfide gas, such as different wind speed, hydrogen sulfide concentration, lye concentration, and spraying amount, were tested. The order of influence factors of H_2_S by spraying absorbing solution was as follows: wind speed in pipeline > mass fraction of alkaline solution > hydrogen sulfide concentration > spray flow rate.

**Figure 16:**
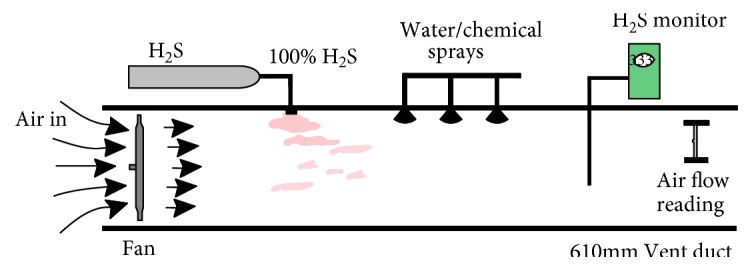
Setup for H_2_S spray tests.

Harvey et al. [26] built a H_2_S spray test system, Figure 16, to simulate a H_2_S contaminated mine roadway and to test the effects of water, different chemicals, varying pH, varying flow rates, and varying spray droplet size, with the best giving a 91 percent reduction in H_2_S levels. The initial test used sodium hydroxide to control pH and sodium hypochlorite to oxidize H_2_S. This test proved to be effective; however, the pH of the solution at 12.4 was unacceptable for the mining environment. Varying spray droplet size between 50 and 150 micron produced little difference in the effective removal of H_2_S.

### 2.3. Hydrogen Sulfide Prevention and Control Technology in Groundwater

H_2_S has a high solubility in water, and its solubility in water is 93 times that of CH_4_ and 2.7 times that of CO_2_. Guang'an Coal Mine in the Huayingshan Coalfield of Sichuan province, during the construction of the North West Wing Station at the level of +497.5 m, penetrated the hydrogen sulfide-containing water with a maximum flow of 40 m^3^/h and a concentration of 180 mg/L. The H_2_S concentration in the surface air is as high as 0.7%. By adopting series ventilation, increasing the air volume, and assisting the treatment of lime in the underground, the comprehensive treatment of hydrogen sulfide water, such as plugging, sparse, and negative pressure ventilation [27, 28], has achieved good effect.

**Figure 17:**
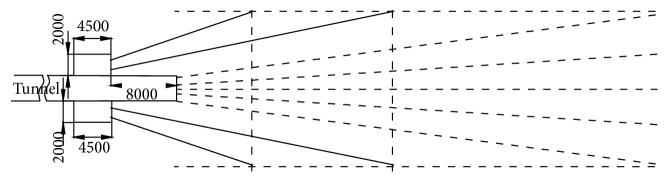
Schematic diagram of tapered curtain drilling arrangement design.

The Binlang Coal Mine in Sichuan province [28], encountered water inrush at the ±0 m horizontal cross cut tunneling accompanied by a high-sulfur-bearing hydrogen reservoir from the Leikoupo formation. The gas emission is stable at around 2 m^3^/min. The CH_4_ concentration in transportation gate is up to 43%, the H_2_S concentration is up to 240 ppm, and the water inrush at the water inrush point is 105 m^3^/h. It has characteristics of abundant gas supply and large water inflow, through adopting long pumping and long pressure ventilation, combined with guiding and isolating drainage, adopting 3% ~ 5% sodium carbonate solution spray method to absorb H_2_S in air, and adopting full-section curtain pregrouting for hydrogen sulfide gushing roadway section. Through comprehensive management, the hydrogen sulfide concentration reduced to 6 ppm. The schematic diagram of tapered curtain drilling arrangement design is shown in Figure 17 [28].

Lin Hai et al. [29] treated the aqueous solution containing H_2_S with a concentration of 140 mg/L by using Fenton reagent with a concentration ratio of 0.67 g/L of FeSO_4_•7H_2_O + 0.67 mL/L of H_2_O_2_. The optimum pH was 6 ~ 10, with the most suitable being 8; when the medium temperature is 25°C, the reaction time is 10 min; the maximum removal rate of H_2_S in the water to the agent is 93.14%.

Wang Xiaojun et al. [30] invented a hydrogen sulfide treatment device for underground water outlets. By designing an automatic spray device for quantitative dosing and absorption of powder, the integrated treatment of hydrogen sulfide at the gushing point was realized.

## 3. Major Problems Existing

### 3.1. Hydrogen Sulfide in Coal-Bearing Formations

Although the method of using alkaline chemicals to treat hydrogen sulfide in coal-bearing formations is an active prevention and control measure, due to the large amount of some chemical agents, the treatment cost is high, and there is a possibility of causing secondary pollution and equipment corrosion [31–33]. And the neutralization reaction of the commonly used sodium carbonate and sodium hydrogen carbonate solution to absorb hydrogen sulfide is reversible, the sodium hydrosulfide formed by the reaction is a chemically unstable compound, the concentration of the sodium carbonate solution is low, and the generated sodium hydrosulfide, in turn, reacts with carbon dioxide to liberate hydrogen sulfide gas. The sodium bicarbonate absorption effect of hydrogen sulfide is not as good as that of sodium carbonate, which reduces the absorption effect of hydrogen sulfide. The addition of oxidants or surfactants tends to result in a doubling of costs, and some strong oxidants are hazardous to the environment and equipment. The use of advanced drilling lye can often affect the speed of excavation.

### 3.2. Hydrogen Sulfide in Roadway Airflow

Strengthening ventilation can only play a role in dilution when the volume fraction of H_2_S is low. For a higher volume fraction of H_2_S, if it is to be reduced below the safe value, the air volume often needs to be doubled or even increased by a factor of ten, so uneconomical ventilation may also cause wind speed and wind pressure to be too high, which is technically and economically unfeasible. The excavation working face adopts the method of changing the ventilation method to treat hydrogen sulfide. It cannot also guarantee that all workers are not harmed by H_2_S. Spraying alkali liquor in the roadway airflow is a passive prevention method, which can only solve the problem of the hydrogen sulfide that has been poured out temporarily; hydrogen sulfide cannot be controlled fundamentally.

### 3.3. Hydrogen Sulfide in Groundwater

The hydrogen sulfide that emission along with water body often has gas and hydrogen sulfide together emission in violent form; gas emission quantity is big and has the characteristic of long-term stability. To deal with this type of hydrogen sulfide, it is often necessary to simultaneously control the problem of water inrush, so its prevention and control are also complicated and difficult.

### 3.4. Other Issues

Hydrogen sulfide is a polar molecule [34, 35]. Coal rock has the characteristics of adsorption and molecular screening. The H_2_S polarizability is 3.64 × 10^-30 ^m^3^ (CH_4_ is 2.60 × 10^-30 ^m^3^). The higher the polarizability, the larger the molecular deformation. Under the same geological conditions, the larger the polarizability is, the easier the molecules are adsorbed. The adsorption capacity of coal for gas increases with the increase of boiling point of gas. The boiling points of H_2_S, CO_2_, CH_4_, and N_2_ are −60.33°C, -78.50°C, -161.49°C, and -195.8°C. It is known that coal on the gas adsorption ability size order is H_2_S > CO_2_ > CH_4_ > N_2_. It can be seen that H_2_S in the coal rock layer is mainly in the state of adsorption, and its adsorption capacity is very strong. Therefore, the use of gas extraction or injection of carbon dioxide to extract H_2_S often leads to the inability to completely cure hydrogen sulfide and is likely to cause environmental pollution and equipment corrosion.

## 4. Research Results

**Figure 18:**
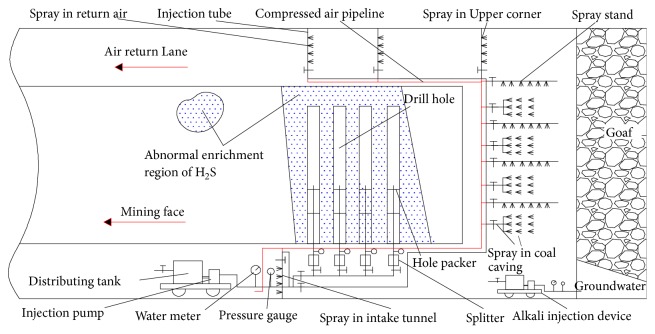
Scheme of comprehensive prevention and control technology of H_2_S on a longwall face in coal mine.

H_2_S needs to be able to survive and be enriched under special geological conditions. In the process of coal mine excavation with abnormal hydrogen sulfide, the hydrogen sulfide enriched in the coal stratum will rush out (escape) into the roadway or mining face, a large amount of hydrogen sulfide escapes during the crushing process, hydrogen sulfide is often emitted from the upper corners and goafs, and a large amount of hydrogen sulfide is often enriched in the water. Furthermore, it brings great difficulty to the prevention and control of hydrogen sulfide in coal and rock layers. Therefore, according to the actual conditions of the mine, each coal mine often needs to establish a comprehensive prevention and control technical scheme of hydrogen sulfide, including “removing, discharging, plugging, sparse and pumping”, as shown in Figure 18 [25].

Removing: It is mainly carried out using chemical means, using spray, sprinkle, and injection (alkali, buffer solution, and surfactant) to neutralize hydrogen sulfide in air, coal rock formations, or water. For the anomalous area of hydrogen sulfide in the coal stratum, the hydrogen sulfide in the coal seam is neutralized by injecting an alkaline solution (or containing an additive or a surfactant) by static pressure or dynamic pressure by drilling in an abnormal region. For coal seams with poor permeability, the local block section can be combined with hydraulic fracturing, loose blasting, and other antireflection measures to improve the injection effect and penetration range.

Discharging: In the case of a small concentration of hydrogen sulfide in the tunnel airflow, H_2_S is discharged or diluted by changing the ventilation method, coal mining method, or increasing the ventilation amount. Or a special return airway is established, the H_2_S-containing gas is led to the special return roadway, and some blocks can use a special diluter.

Plugging: For the mining area similar to the Huayingshan mining area in Guangyuan, Sichuan province, the H_2_S brought by the water body can be combined with plugging and sparse methods, mainly adopting grouting method and principle, using slurry filling or infiltration to seal the crack and isolate the water source, thereby plugging and to sparse the water containing hydrogen sulfide. The sealing material can be selected from barite powder, bentonite, sodium carboxymethyl cellulose (CMC) binder, Na_2_CO_3_, NaOH, solid plugging material, cement, and quick-setting admixture. For conditions similar to those of Xishan Coal Mine in Wulumuqi, Xinjiang, although the amount of water in each place of the roadway is not large, the water body is rich in hydrogen sulfide, and the lime water block or lye can be regularly sprayed to the gushing port for auxiliary prevention and control. For hydrogen sulfide that is vented through the roadway crack, high pressure grouting can be used to seal the crack, forcing the hydrogen sulfide to leak.

Sparse: For hydrogen sulfide generated by water bodies similar to the Huayingshan mining area in Guangyuan, Sichuan, a special pipe network can be used to drain the hydrogen sulfide-containing water to a designated location on the basis of plugging, and then chemical agents are used for prevention and control.

Pumping: In the drainage method, for areas with large H_2_S content per ton of coal, special pipe network can be used to extract hydrogen sulfide from the coal seam by pressure difference.

**Figure 19:**
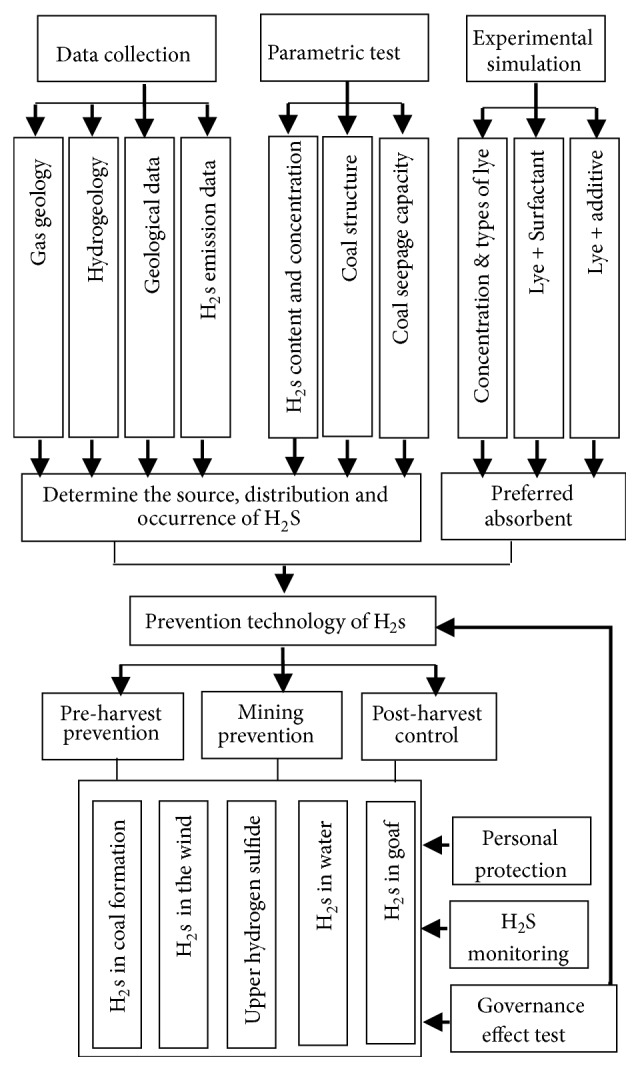
Complete set of technical systems of comprehensive prevention and control technology of H_2_S in coal mine.

Each mine should combine the hydrogen sulfide distribution and gushing characteristics of the mine and combine the coal mining order to establish a complete technical system for comprehensive control of coal sulfide hydrogen sulfide as shown in Figure 19 [35].

## 5. Conclusions and Prospects

(1) The abnormal gushing and injury accidents caused by high hydrogen sulfide gas in coal mines are becoming more and more frequent, and disaster prevention and control will be paid more and more attention. The characteristics of hydrogen sulfide determine its heterogeneity in the distribution of coal and rock layers, and the zoning phenomenon is obvious. Therefore, before the prevention and control of hydrogen sulfide in coal mines, it is necessary to find out the occurrence, distribution characteristics, and content of coal sulfide in coal seams and carry out targeted prevention and control on this basis.

(2) The alkali concentration ratio of hydrogen sulfide in the coal seam should be calculated based on the H_2_S content in ton coal. In the specific prevention and control process, the ratio of the alkali solution should be adjusted according to the H_2_S content of the coal seam to ensure the best treatment effect. In the lye ratio, it is necessary to comprehensively consider the drilling depth, spacing, coal seam permeability coefficient, coal seam thickness, working surface length, water loss, water injection wet radius, water injection unevenness coefficient, water injection pressure, and water injection time.

(3) At present, the majority of coal mine hydrogen sulfide treatment technologies use local control measures. Although some effects have been achieved, there is still a big gap between safe and efficient mining of coal mines. Each mine should combine the specific conditions of the mine, increase basic experimental research, screen economic and high-efficiency hydrogen sulfide prevention and control agents, and establish a comprehensive and long-term prevention and control technical measures.

## Figures and Tables

**Table 1:** The environmental standards of H_2_S of difference control.

Characteristic boundary		Gas concentration	Source
Smell	Stimulus threshold	0.012 mg/m^3^ ~ 0.03 mg/m^3^ (0.007 5 mg/m^3^ ~ 0.019 mg/m^3^)	Hygiene (Fifth Edition)
	Moderate odor	3 ppm ~ 4.60 ppm	

Health standard	Workshop	10 mg/m^3^ (6.5 ppm)	≪TJ36-79 Industrial Enterprise Design Hygiene Standards≫
	Residential area	0.01 mg/m^3^ (0.0065 ppm)	
	Maximum allowable concentration in the workplace (MAC)	10 mg/m^3^ (6.5 ppm)	≪GBZ2-2002 Occupational Exposure Limits for Harmful Factors in the Workplace≫
	Coal mine	6.6 ppm	≪Coal Mine Safety Regulations≫
	America	10 ppm (15min)	US Health Authority (1975)
	Japan	10 ppm (15min)	Japan Industrial Hygiene Society (1975)

**Table 2:** Sources of H_2_S during mining.

Source	Range
Shearer	50% to 70% [3–5]

Armoured face conveyor (depends on location of H_2_S zone along face)	5% to 10% [3–5]

Maingate corner	10% to 15% [3–5]

Desorbed from face	5% to 10% [3–5]

Crusher, Beam stage loader and longwall conveyor	10% to 20% [3–5]
